# The Genome Sequence of *Bifidobacterium moukalabense* DSM 27321 Highlights the Close Phylogenetic Relatedness with the *Bifidobacterium dentium* Taxon

**DOI:** 10.1128/genomeA.00048-14

**Published:** 2014-02-20

**Authors:** Gabriele Andrea Lugli, Sabrina Duranti, Christian Milani, Francesca Turroni, Alice Viappiani, Marta Mangifesta, Douwe van Sinderen, Marco Ventura

**Affiliations:** a Laboratory of Probiogenomics, Department of Life Sciences, University of Parma, Parma, Italy; b GenProbio, Parma, Italy; c Alimentary Pharmabiotic Centre and Department of Microbiology, Bioscience Institute, National University of Ireland, Cork, Ireland

## Abstract

*Bifidobacterium moukalabense* DSM 27321 is the reference strain for a recently described new bifidobacterial species that has been isolated from a wild west lowland gorilla. Here, we report the whole-genome sequence of DSM 27321, which supports very close phylogenetic relatedness with members of the *Bifidobacterium adolescentis* phylogenetic group and, in particular, the *Bifidobacterium dentium* taxon.

## GENOME ANNOUNCEMENT

Bifidobacteria have been extensively detected in the gut of mammals, birds, and social insects (1). Genome sequencing has specifically applied to this group of microorganisms and allowed the identification of the genetic determinants sustaining the adaptation to specific ecological niches (2, 3). In addition, bifidobacterial genomics is an important discovery approach to reveal how they are phylogenetic related and how a newly identified bifidobacterial taxon, such as *Bifidobacterium moukalabense*, is genetically related to the other members of the genus *Bifidobacterium*.

Here, we describe the draft genome sequence of the type strain, i.e., DSM 27321, of the recently described *B. moukalabense* species (4). This species has been reported to belong to the *Bifidobacterium adolescentis* phylogenetic group, which currently consists of *B. adolescentis*, *Bifidobacterium catenulatum*, *Bifidobacterium pseudocatenulatum*, *Bifidobacterium ruminantium*, and *Bifidobacterium dentium* (5). The complete genome sequence of DSM 27321 was determined using cells from the DSMZ bacterial culture collection. The genome sequence of DSM 27321 was determined by GenProbio srl using Ion Torrent PGM (Life Technologies). The generated sequences represented an 81.76-fold coverage of the *B. moukalabense* DSM 27321 genome and were assembled into 12 contigs to yield a consensus sequence of 2,515,335 bp with a GC content of 59.87%, which is almost identical to that of the *B. dentium* Bd1 genome (6). The DSM 27321 genome contains 2,046 open reading frames (ORFs), and it possesses 57 tRNAs and 4 rRNA operons. This overall genome structure is very similar to that identified in other members of the *B. adolescentis* phylogenetic group chromosomes (7).

The genome structure of DSM 27321 is highly syntenic with that of the recently sequenced genome of *B. dentium* Bd1, with an average nucleotide identity of 78.02% across these two genomes as determined by the use of a Stretcher alignment (8). The very close phylogenetic relatedness between these two strains was also confirmed by phylogenomic analyses based on the core gene sets that have recently been described as a valid reference database for analyzing the genomic variability within the *B. adolescentis* phylogenetic group (7).

All together, these analyses are indicative of a monomorphic genomic structure of *B. moukalabense* and *B. dentium* showing that the strains DSM 27321 and Bd1 have a very close isogenic nature. Furthermore, our analyses suggest that the *B. moukalabense* species should be considered a subjective synonym of the *B. dentium* taxon.

### Nucleotide sequence accession numbers.

This whole-genome shotgun project has been deposited at DDBJ/EMBL/GenBank under the accession number AZMV00000000. The version described in this paper is version AZMV01000000.
